# Spinal gastrin releasing peptide receptor expressing interneurons are controlled by local phasic and tonic inhibition

**DOI:** 10.1038/s41598-019-52642-3

**Published:** 2019-11-12

**Authors:** Fabio B. Freitag, Aikeremu Ahemaiti, Jon E. T. Jakobsson, Hannah M. Weman, Malin C. Lagerström

**Affiliations:** Department of Neuroscience, Uppsala University, Uppsala, Sweden

**Keywords:** Neural circuits, Sensory processing

## Abstract

Dorsal horn gastrin-releasing peptide receptor (GRPR) neurons have a central role in itch transmission. Itch signaling has been suggested to be controlled by an inhibitory network in the spinal dorsal horn, as increased scratching behavior can be induced by pharmacological disinhibition or ablation of inhibitory interneurons, but the direct influence of the inhibitory tone on the GRPR neurons in the itch pathway have not been explored. Here we have investigated spinal GRPR neurons through *in vitro* and bioinformatical analysis. Electrophysiological recordings revealed that GRPR neurons receive local spontaneous excitatory inputs transmitted by glutamate and inhibitory inputs by glycine and GABA, which were transmitted either by separate glycinergic and GABAergic synapses or by glycine and GABA co-releasing synapses. Additionally, all GRPR neurons received both glycine- and GABA-induced tonic currents. The findings show a complex inhibitory network, composed of synaptic and tonic currents that gates the excitability of GRPR neurons, which provides direct evidence for the existence of an inhibitory tone controlling spontaneous discharge in an itch-related neuronal network in the spinal cord. Finally, calcium imaging revealed increased levels of neuronal activity in Grpr-Cre neurons upon application of somatostatin, which provides direct *in vitro* evidence for disinhibition of these dorsal horn interneurons.

## Introduction

Itch was defined in the 17^th^ century by Samuel Hafenreffer as an “unpleasant sensation that elicits the desire or reflex to scratch”^1^. Itch-related information is transmitted from the skin to the dorsal horn of the spinal cord by slow unmyelinated C-fibers^2,3^. Sun and Chen reported the first evidence for a labeled line of itch in the spinal cord in 2007, where the gastrin-releasing peptide (GRP) and its receptor GRPR form two parts of a system directly related to itch but not pain^4^. Compared to control animals, decreased scratching behavior could be observed in *Grpr* null mice when scratching was induced intradermally by the pruritogenic agent 48/80 or intrathecally by GRP. Reduced scratch behavior was also reported when the vesicular glutamate transporter 2 (VGLUT2) was conditionally removed from the Grpr-Cre population^5^.

Itch can also be induced when the inhibitory tone in the dorsal horn is impaired. Accordingly, transgenic mice lacking inhibitory interneurons marked by the transcription factor Bhlhb5 developed a spontaneous scratch behavior and heightened responses to pruritogenic stimuli^6^. A similar effect was observed when the somatostatin receptor sst_2A_ agonist octreotide was injected intrathecally^7^, which suggested that itch was induced by a disinhibition process as activation of sst_2A_ causes hyperpolarization and sst_2A_ is present in mainly inhibitory spinal interneurons^8^. Finally, ablation of inhibitory glycinergic spinal interneurons induced a spontaneous aversive behavior whereas activation was shown to decrease scratch behavior induced by histamine and chloroquine^9^. Based on these findings, the inhibitory tone in the dorsal horn may be an important mechanism to regulate the sensation of itch.

Inhibition can be mediated by two pathways: short lasting inhibitory postsynaptic current (IPSC) via synaptic receptors, and long lasting inhibitory currents via peri-/extra-synaptic receptors referred to as tonic currents^10^. In contrast to short lasting synaptic inhibition, tonic currents generate persistent inhibition that continuously hyperpolarizes the postsynaptic membrane, which acts as a gate control to regulate the excitability of the neuron^11^. Tonic currents have been identified in the spinal cord^12–14^ but the presence of tonic inhibition on specific sensory populations in the spinal cord has not been investigated.

Recently, Huang and co-workers showed through a set of pharmacological experiments that itch induced by a disinhibition process is linked to the GRP/GRPR system^15^. They showed that GRP increases the level of scratch behavior induced by octreotide, and conversely, that GRP-induced scratch behavior was reduced when GRP was co-injected with the sst_2A_ receptor antagonist CYN154086^15^. Also, a decrease in octreotide-induced scratch behavior was observed in GRP saporin-treated mice and GRP antagonist-injected animals^15^, indicating that GRPR neurons are involved in disinhibition-induced itch. Here we have used the Grpr-Cre transgenic mouse line^5^, in combination with whole cell patch clamp recordings, AAV9.GCaMP6-enabled calcium imaging and bioinformatics, to investigate the direct regulatory inputs to GRPR dorsal horn interneurons *in vitro*, and to test on a cellular level if the activity of this neuronal population is controlled by local inhibition to prevent spontaneous activity in itch-related neuronal networks.

## Results

### Adult Grpr-Cre neurons are mainly excitatory but a small inhibitory population is also present in the spinal cord dorsal horn

**Figure 1:**
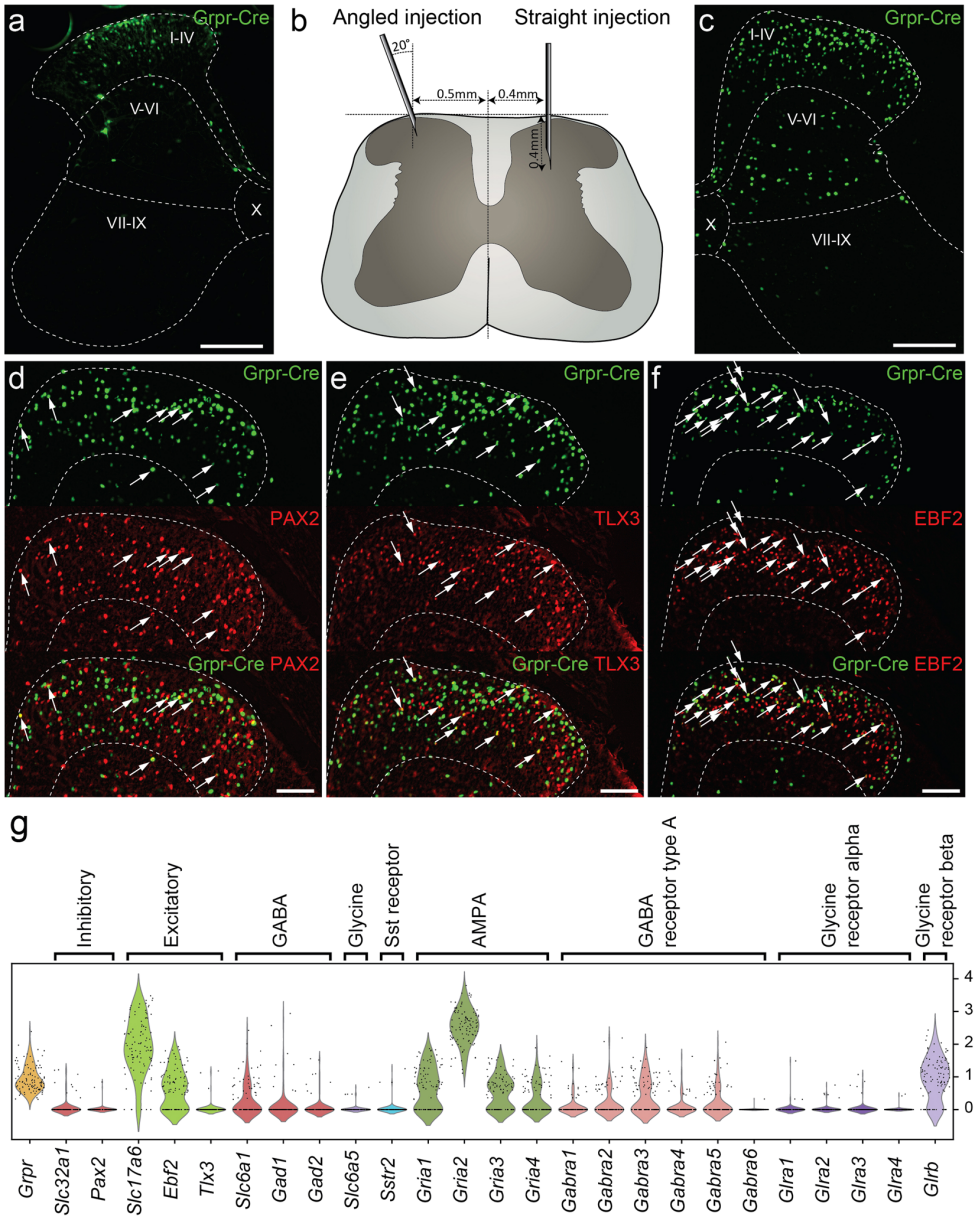
The Grpr-Cre population of spinal interneurons is predominantly excitatory. Analysis of adult Grpr-Cre neurons displayed through a viral reporter system. (**a**) Coronal section of lumbar spinal cord annotated using Rexed laminae following an injection of AAV2-EF1a-DIO-EYFP virus using the angled injection scheme. Grpr-Cre neurons in green. (**b**) Illustrating the difference between the angled and the straight injection schemes. (**c**) Coronal section of lumbar spinal cord following an injection of the reporter virus AAVDJ-EF1a-DIO-HTB using the straight injection scheme. Grpr-Cre neurons in green. (**d–f**) Immunohistochemistry against PAX2 (d), TLX3 (e) and EBF2 (f) with Grpr-Cre neurons in green and the antigen in red. Dashed lines outline lamina I-IV. Arrows indicate Grpr-Cre neurons overlapping with the antigen. (**g**) *Grpr* neurons express both excitatory and inhibitory markers and genes important for glutamatergic, GABAergic and glycinergic signaling input. Violin plot of normalized expression of marker genes and AMPA, GABA receptor type A and glycine receptor subunits in *Grpr*-expressing neurons. For gene expression of all presented genes in all Häring cell types see Figure S3A. For gene expression in all *Grpr* neurons grouped by Häring cell types, see Figure S3B. Scale bars in (**a**) and (**c**) correspond to 200 µm while scale bars in (**d–f**) correspond to 100 µm.

To study the neuronal network that regulate the activity of GRPR neurons in the spinal cord, we first set out to characterize the neurochemical identity of the adult neuronal population. We have previously shown that the Grpr-Cre population of spinal interneurons comprises *Grpr* mRNA expressing neurons and marks an extended population during development^5^. We have also shown that all tested adult virally marked Grpr-Cre cells respond to GRP^5^, so to characterize only the adult Grpr-Cre cells, AAVDJ-EF1a-DIO-HTB virus was injected into the lumbar spinal cord to conditionally mark the adult Grpr-Cre neurons. Viral injections were made using a 20° angled injection (Fig. 1a,b), according to^16^, or through a straight non-angled injection (Fig. 1b,c). A comprehensive and consistent labeling of the Grpr-Cre population was obtained using a straight injection, hence this injection scheme was subsequently used. Virus-infected cells were found in both the dorsal and ventral horn, with the majority (91.8 ± 0.5%, n = 3 animals) found in the dorsal laminae (I-VI) (Fig. 1c). Inhibitory neurons were labeled using an antibody against Paired box gene 2 (PAX2) (Fig. 1d), which has been shown to be expressed in most GABAergic and glycinergic neurons throughout the dorsal horn^17^. In total, 13.5 ± 0.5% of lamina I-IV Grpr-Cre neurons were PAX2 positive (n = 3 animals, 40 sections), indicating that a small population of adult Grpr-Cre neurons are inhibitory. To further validate the finding, PAX2 immunohistochemistry was performed on sections from vesicular inhibitory amino acid transporter (VIAAT)-EGFP spinal tissue, where 84.6 ± 1.7% of all VIAAT positive cells were found to be PAX2 positive (Figure S1a,b).

Immunohistochemical markers for excitatory neurons in the adult dorsal horn are not as well established as PAX2 is for inhibitory neurons. However, TLX3 is a transcription factor important for the development of excitatory neurons in the superficial dorsal horn, with both transient expression during development and persistent expression in a subpopulation of excitatory neurons in adults^18^. The overlap analysis showed that 28.5 ± 0.2% of lamina I-IV Grpr-Cre neurons were TLX3 positive (n = 3 animals, 42 sections) (Fig. 1e). Furthermore, EBF2, a neuronal marker with broad expression patterns among the excitatory neurons in the superficial dorsal horn^8,19^ (74.1% of *Slc17a6* (VGLUT2)–expressing neurons contain *Ebf2*^8^), showed a 59.2 ± 3.7% overlap with lamina I-IV Grpr-Cre neurons (n = 3 animals, 35 sections) (Fig. 1f). In comparison, a bioinformatical analysis of all spinal *Grpr*-expressing neurons in the Häring *et al*. single-cell mRNA sequencing (scRNA-seq) dataset (genes prevalent if log1p > 0.01), showed that the *Grpr* population has low expression of *Tlx3* (2.9% of *Grpr* neurons express *Tlx3*), moderate expression of *Ebf2* (64.1% of *Grpr* neurons express *Ebf2*) and high expression of *Slc17a6* (*Vglut2)* (94.2% of *Grpr* neurons express *Slc17a6*). Regarding inhibitory markers, low expression of *Slc32a1* (*Vgat/Viaat*) (8.7% of *Grpr* neurons express *Slc32a1*) and *Pax2* (1.9% of *Grpr* neurons express *Pax2*) was also found (Table S1, Fig. 1g). In conclusion, the adult spinal Grpr-Cre population consists of mainly excitatory neurons and a small inhibitory subpopulation, which is in agreement with the bioinformatical findings.

### GRPR neurons receive spontaneous excitatory and inhibitory synaptic inputs, but also extra-synaptic tonic inhibitory inputs

**Figure 2:**
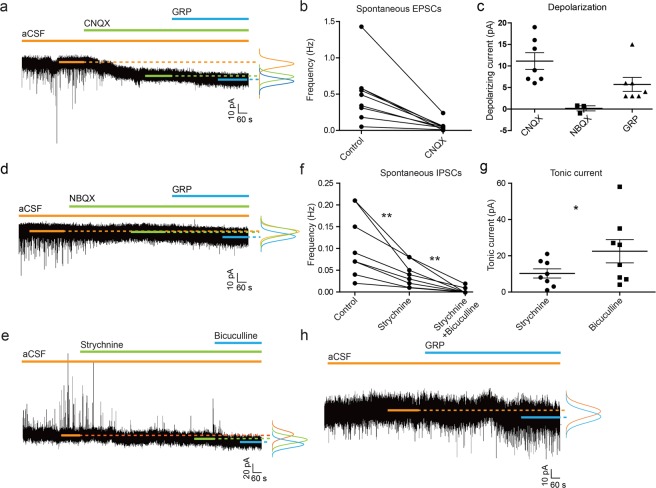
GRPR neurons receive spontaneous excitatory input via glutamate and inhibitory inputs via both glycine and GABA. (**a)** sEPSCs were blocked by CNQX, which also induced depolarization. GRP was applied at the end of each recording to confirm the expression of GRPR on the recorded cell and the effect was determined by the depolarization of the membrane upon application. The bell-curve represents the conventional distribution histogram of the data points at the selected region and the peak of the curve indicates the holding current. (**b**) The amount of excitatory synaptic inputs was reduced by CNQX, showing that the sEPSCs were mainly mediated by AMPA. (**c**) The depolarizing current induced by CNQX, NBQX and GRP. (**d**) The AMPA receptor antagonist NBQX did not induce any depolarization. (**e**) sIPSCs were mostly blocked by glycine receptor antagonist strychnine and the remaining currents were blocked by GABA receptor antagonist bicuculline, which was applied in the presence of strychnine. Application of strychnine and bicuculline revealed the glycine- and GABA-induced inhibitory tonic current respectively. (**f**) The reduction of sIPSCs upon application of strychnine (62%) (n = 8, two-tailed T-test, p = 0.0076) and bicuculline (38%) (n = 8, two-tailed T-test, p = 0.0046) is shown as a reduction in sIPSCs frequency. (**g**) GABA induced significantly larger tonic current than glycine (revealed by the application of respective antagonist) (n = 8, two-tailed T-test, p = 0.047). (**h**) GRP confirmed the expression of GRPR by inducing depolarization.

After histological characterization of the adult Grpr-Cre dorsal horn population, we aimed at exploring its local input connections using whole cell patch clamp recordings to identify how the activity of these neurons is regulated. Here, we focused on the main classical neurotransmitters in the spinal cord i.e. glutamate, GABA and glycine. The whole cell patch clamp analysis on Grpr-Cre;*tdTomato* neurons showed that the excitatory inputs were predominantly mediated by AMPA (α-amino-3-hydroxy-5-methyl-4-isoxazolepropionic acid) receptors, as about 90% of the spontaneous EPSCs were blocked by the AMPA receptor antagonist CNQX (Fig. 2a,b). In addition to its blocking effect on EPSCs, CNQX also induced depolarization (11.5 ± 4.8 pA, Fig. 2c). The depolarizing effect of CNQX has also been shown in different brain regions^20,21^. Therefore, the depolarization could be due to the direct effect of CNQX on the patched neuron or through disinhibition via reducing the excitability of the presynaptic inhibitory neuron by blocking the EPSCs on the presynaptic neuron. To clarify this, an alternative AMPA receptor antagonist NBQX, which does not induce depolarization^21^, was also tested. NBQX blocked EPSCs but did not induce any depolarization (Fig. 2c,d), showing that the depolarizing effect of CNQX was not through an altered inhibitory pathway. GRP was applied at the end of every recording as a positive control of the adult expression of GRPR as Grpr-Cre;*tdTomato* marks an extended population during development (Aresh *et al*., 2017, Pain), and only Grpr-Cre;*tdTomato* cells that were depolarized upon application of GRP (10 out of 17 neurons, 59%) (Fig. 2a–d) were considered as GRPR neurons and included in the statistics.

Further analysis of the GRPR positive neurons showed that GRPR neurons also receive sIPSCs. Application of strychnine (a glycine receptor antagonist) removed 62% of the sIPSCs and the remaining 38% of the sIPSCs were blocked by the application of bicuculline (a GABA receptor antagonist) in the presence of strychnine (Fig. 2e,f). By analyzing the kinetics of the sIPSCs and their response to the antagonists, we found that 34% of the sIPSCs were mediated by fast-decaying glycinergic receptors (decay time constant of 6.2 ± 2.2 ms), 25% by slow-decaying GABAergic receptors (decay time constant of 42 ms), and 41% of the sIPSCs displayed intermediate decay time constant that ranged between 16.8–35.7 ms, which corresponded to glycine and GABA co-released events^22^.

**Figure 3:**
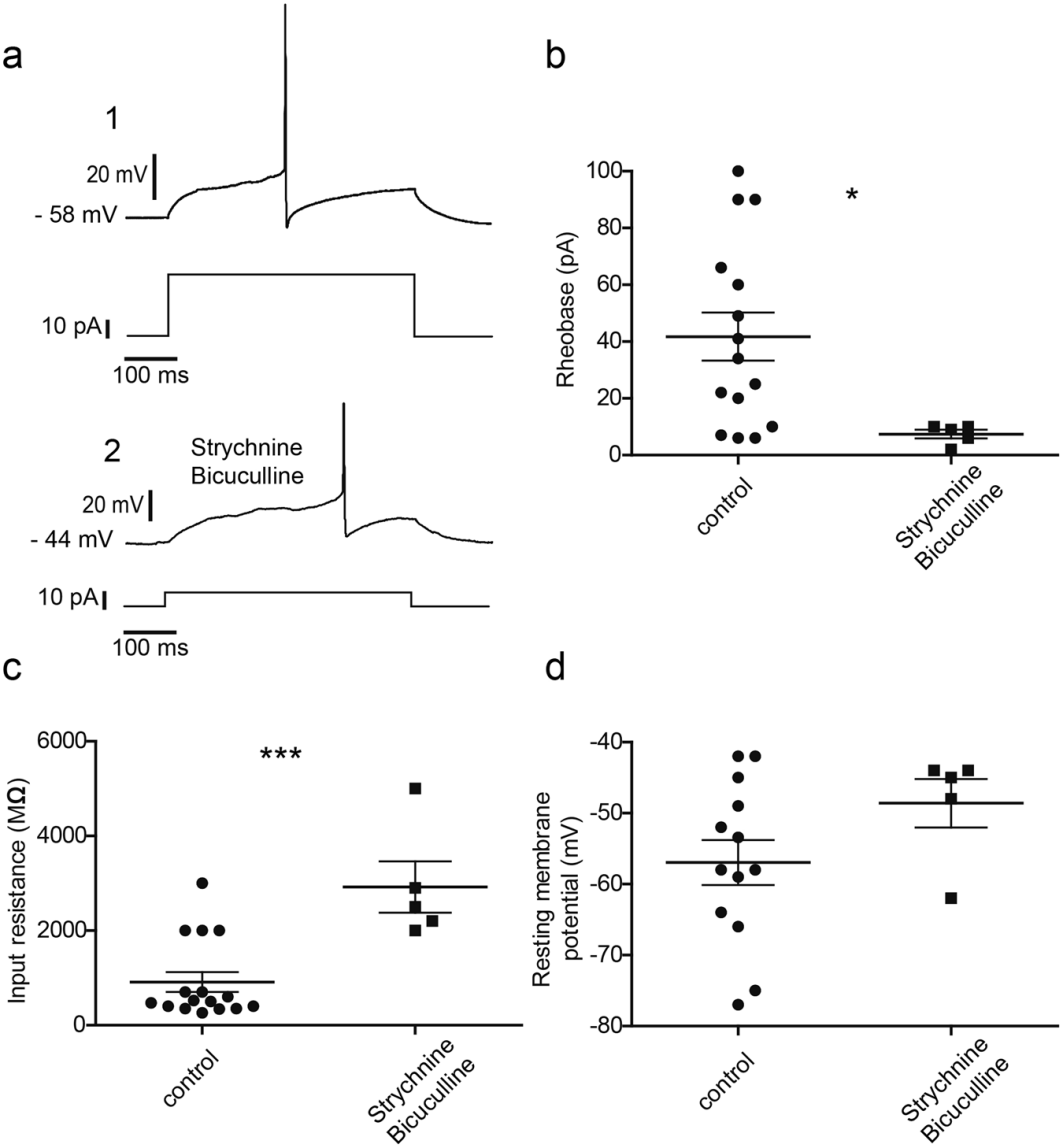
Antagonizing the glycine and GABA receptors increased the excitability of the GRPR neurons. (**a**) Current induced action potentials from GRPR neurons. The upper trace in **a1** is the induced action potential and the lower trace is the stimulating current pulse (duration 500 ms). **a2** shows the induced action potential after strychnine and bicuculline treatment. (**b**) Comparison of the rheobase between control group (n = 15) and the antagonist treated neurons (n = 5) (two-tailed T-test, p = 0.0337). (**c**) Input resistance comparison between control group (n = 16) and the antagonist treated neurons (n = 5) (two-tailed T-test, p = 0.0005). (**d**) Resting membrane potentials in the control group (n = 13) and the antagonist treated neurons (n = 5).

In addition to the synaptic transient current, all GRPR neurons displayed both glycine- and GABA-induced tonic inhibitory currents (10.25 ± 6.7 pA and 22.5 ± 17 pA respectively, Fig. 2g). Among all the sIPSCs-recorded Grpr-Cre;*tdTomato* neurons, about 62% (8 out of 13) responded to GRP, which is consistent with previous findings (Fig. 2h)^5^. Additionally, antagonizing the inhibitory inputs by strychnine and bicuculline significantly increased the excitability of the GRPR neurons (Fig. 3a,b). Blocking of the glycine and GABA receptors significantly reduced rheobase (39.8 ± 8.2 pA (control) and 7.4 ± 1.5 pA (after antagonist treatment)) (Fig. 3b). The input resistance also showed a significant increase (911.9 ± 209.4 MΩ (control) and 2920 ± 541.7 MΩ (after antagonist treatment)) (Fig. 3c). Although not statistically significant, the mean resting membrane potential showed depolarization after glycine and GABA receptor blocking (−56.96 ± 3.16 mV (control) and 48.6 ± 3.43 mV (after antagonist treatment)) (Fig. 3d).

Spinal inhibitory interneurons control the tone in somatosensory systems to prevent spontaneous activity and to attenuate inputs to the dorsal horn to attain a suitable level of activation in response to peripheral stimuli^23^. The results presented here demonstrate the existence of a complex local inhibitory tone, composed of both synaptic and tonic currents that specifically regulates the excitability of GRPR neurons, neurons that are known to transmit itch.

### The Grpr population expresses receptor subunits involved in glutamatergic, GABAergic and glycinergic signaling input

To further evaluate our electrophysiological findings from the GRPR population, genes important for GABAergic, glutamatergic and glycinergic signaling input were investigated in the *Grpr*-expressing neurons of the Häring *et al*., 2018 scRNA seq dataset. Transcripts for the AMPA receptor subunits (*Gria1–4)*^24^ were detected in the majority of *Grpr* neurons (*Gria1*: 62.1%, *Gria2: 100%*, *Gria3*: 61.2%), except *Gria4* which was detected in 38.8% of *Grpr* neurons (Fig. 1g, Table S1). Transcripts for the γ-aminobutyric acid A (GABA_A_) receptor subunits (*Gabra1–6*) were less prevalent in *Grpr* neurons compared to the AMPA receptor subunits, with the highest prevalence of *Gabra3* (44.7%), followed by *Gabra5* and *Gabra2* (27.2% and 23.3% respectively). *Gabra1* and *Gabra4* could be detected in 14.6% and 13.6%, respectively, while *Gabra6* was the least prevalent, expressed by only 1% of the *Grpr* neurons. Transcripts for the glycine alpha receptor subunits (*Glra1–4*) were detected in less than 5% of *Grpr* neurons (*Glra1*: 2.9%, *Glra2*: 4.9%, *Glra3*: 4.9%, *Glra4*: 1.9%). However, the *Glrb* receptor subunit was detected in 78.6% of the *Grpr* neurons (Fig. 1g, Table S1). Collectively, the scRNA-seq analysis showed that spinal dorsal horn *Grpr* neurons express genes that code for glutamatergic, GABAergic and glycinergic receptors.

### Action potentials induced by current injections are reflected in increased GCaMP fluorescence

To analyze the *in vitro* consequences of disturbed inhibitory control of the Grpr-Cre population, we continued by analyzing calcium activities in Grpr-Cre neurons in either intact or sliced spinal cord using the calcium-sensitive protein GCaMP6f in control conditions or in the presence of somatostatin, a peptide that has been coupled to increased itch (scratching behavior) through reduced inhibition (disinhibition)^7,15^.

**Figure 4:**
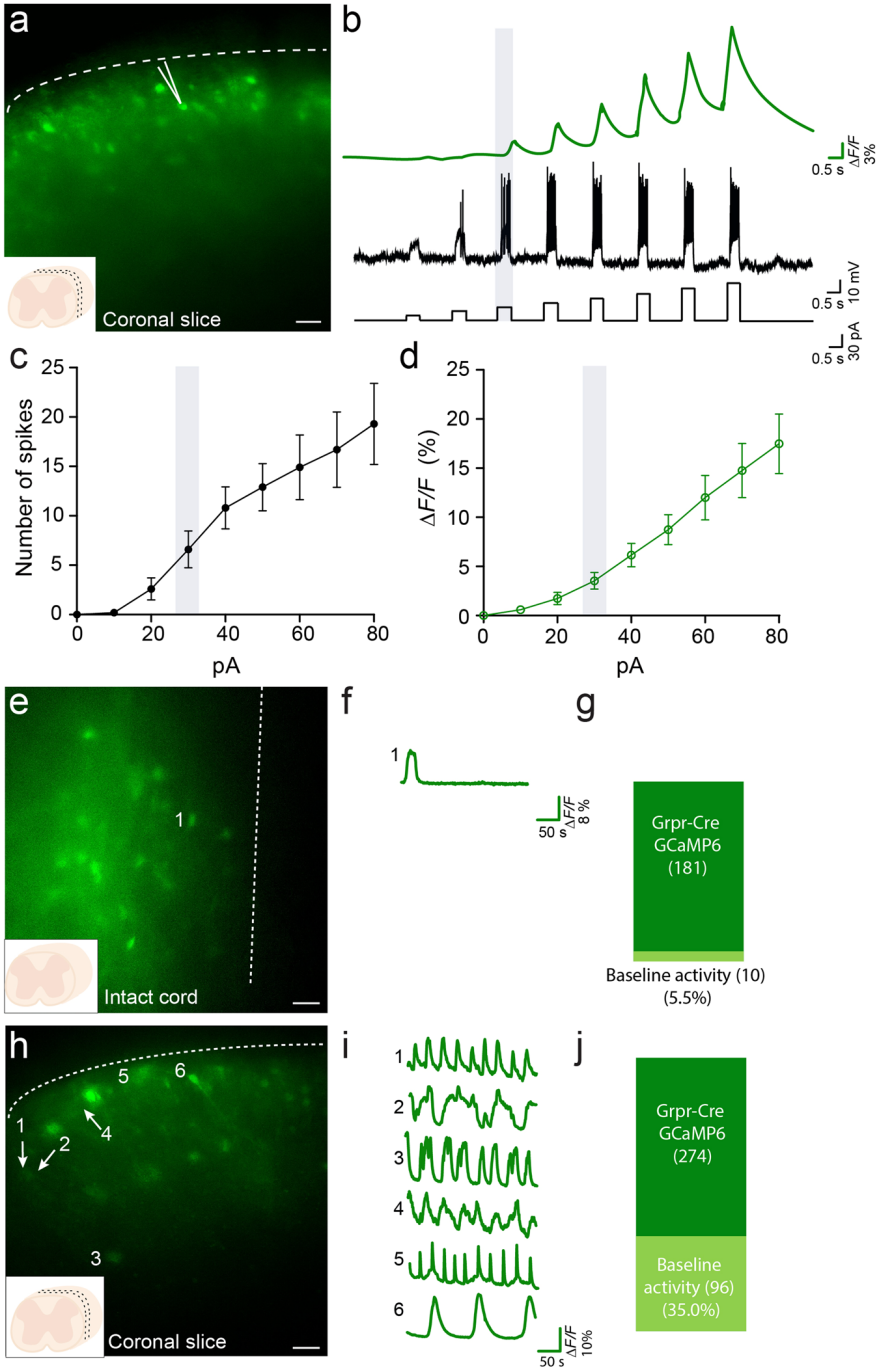
Correlation of induced action potentials and GCaMP6 fluorescence increase based on current injection in whole cell patch clamped neurons. (**a**) Coronal lumbar spinal cord 300 µM slice with Grpr-Cre neurons expressing GCaMP6 from an AAV9-CAG-DIO-GCaMP6 virally injected mouse. Arrow indicates a whole cell patch clamped neuron. Scale bar in A corresponds to 50 µm. (**b**) Fluorescence change and voltage traces from a whole cell patch clamped neuron where a sequence of stepped pulses from 10 pA – 80pA (8 pulses, 10 pA increment, 500 ms pulse duration and 1000 ms pulse interval) was applied. (**c**) Mean number of action potentials (spikes) induced by 8 steps 10 pA increment protocol. (**d**) Mean values for fluorescence increase (∆F/F) induced when recorded neurons were given 10 pA increment protocol in 8 steps. The data is presented as mean ± standard error (n = 10 neurons). The Grpr-Cre population shows basal spontaneous activity in *in vitro* preparations. Example traces from representative samples are shown. (**e**) Grpr-Cre neurons expressing GCaMP6 in an intact spinal cord. Number 1 indicates a neuron showing basal activity under control conditions. (**f**) Fluorescence change trace from the intact spinal cord neuron indicated in (**e–g**) Proportion of Grpr-Cre neurons expressing GCaMP6 in the intact spinal cord that showed basal activity. (**h**) 300 µM coronal slice with Grpr-Cre neurons expressing GCaMP6. Numbers 1–6 indicate six different neurons showing basal activity under control conditions. Numbers 1 and 2 indicate two different neurons that were not active on that specific frame and thus show a low level of fluorescence. (**i**) Fluorescence change traces from the neurons indicated by 1–6 in (**h–j**) Proportion of Grpr-Cre neurons expressing GCaMP6 that showed basal activity in coronal slices. Scale bar in (E, H) corresponds to 40 µm.

In addition to action potential-induced calcium current, subthreshold depolarization of the cell membrane can also induce small, yet detectable, calcium currents. It is thus critical to set a detection threshold for the GCaMP6f calcium signaling to discard calcium signals at subthreshold depolarization levels so that only the action potential-triggered calcium signals are considered in the statistics. To set the threshold, whole cell patch clamped neurons (n = 10 neurons, 2 animals) were kept in a current clamp mode (Fig. 4a) and simultaneously imaged while a sequence of stepped pulses from 10 pA–80 pA (8 pulses, 10 pA increment, 500 ms pulse duration and 1000 ms pulse interval) were delivered (Fig. 4b). Absence of current injection resulted in no change in fluorescence as this set of experiments was done only in non-spontaneously active neurons. A 10 pA current injection induced a 0.6 ± 0.4% increase in the fluorescence and corresponded to 0.2 ± 0.1 induced action potentials, while 30 pA induced a 3.5 ± 0.9% increase in the fluorescence and 6.6 ± 1.9 spikes. An 80 pA current injection induced a 17.5 ± 3.0% increase in the fluorescence and 19.3 ± 4.1 induced spikes (for an overall view on mean values in number of spikes and *ΔF/F*% per current increment steps see Figure 4c,d. Based on this data, we established a threshold of 3.5% in the *ΔF/F*% as indicative of neuronal spiking for further experiments.

### Somatostatin induces activity in Grpr-Cre-expressing neurons

The neurotransmitter somatostatin induces itch through interaction with the spinal inhibitory-coupled sst2a receptor^25^. The sst_2A_ receptor is mainly expressed in inhibitory interneurons in the dorsal horn (Figure S3a)^8^. To further evaluate if Grpr-Cre-expressing neurons could be activated by disruption of local inhibition, somatostatin, was bath applied to either intact spinal cord or coronal slices. This strategy represents a targeted way of studying the effect of attenuated inhibitory input (disinhibition) to the GRPR population as very few *Grpr* neurons (2.9%^8^, Table S1) express the sst_2A_ receptor and therefore an observed change in activity in GRPR neurons, induced by somatostatin, would have risen from changed inhibition from pre-synaptic neurons expressing the sst_2A_.

First, we set out to establish the baseline/control conditions where the tissue was kept in running aCSF. In intact spinal cord, 5.5% of the Grpr-Cre.GCaMP6 expressing neurons showed background/spontaneous activity in the control condition (10 out of 181 neurons, n = 7 animals) (Fig. 4e–g). In coronal slices, 35.0% (96 out of 274 neurons, n = 2 animals) of Grpr-Cre.GCaMP6 neurons showed spontaneous baseline activity under control conditions (Fig. 4h–j) (these levels were comparable to the baseline activity observed in horizontal and sagittal slices (Figure S2)).

**Figure 5:**
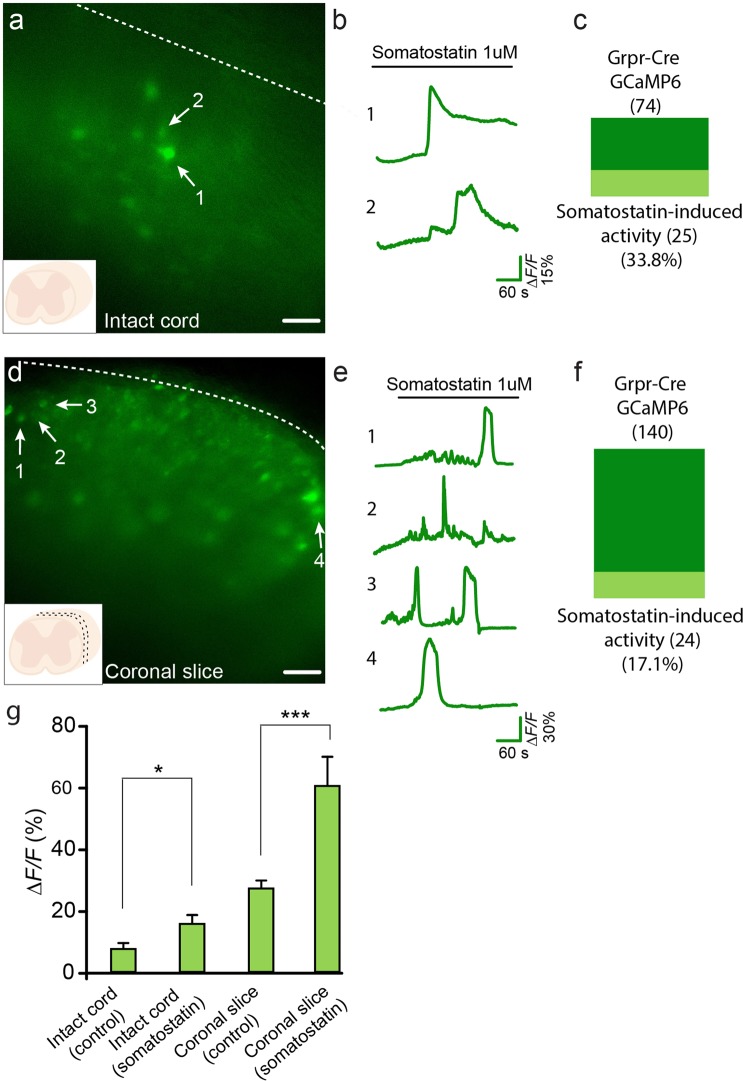
Grpr-Cre neurons expressing GCaMP6 are activated by somatostatin through disinhibition. Example traces from representative samples are shown. (**a**) Grpr-Cre neurons expressing GCaMP6 in an intact spinal cord. Numbers 1 and 2 indicate two neurons showing somatostatin-induced activity. (**b**) Fluorescence change traces from the intact spinal cord neurons indicated in **a**. (**c**) Proportion of Grpr-Cre neurons in the non-spontaneously active population expressing GCaMP6 in intact spinal cord that showed somatostatin-induced activity. (**d**) 300 µM coronal slice with Grpr-Cre neurons expressing GCaMP6. Numbers 1–4 indicate four different neurons showing somatostatin-induced activity. (**e**) Fluorescence change traces from the neurons indicated by 1–4 in (**d–f**) Proportion of Grpr-Cre neurons in the non-spontaneously active population expressing GCaMP6 in coronal slices that showed somatostatin-induced activity. (**g**) Mean values for fluorescence change (*ΔF/F*%) in GCaMP6 intensity for the intact cord and coronal slices, imaged at control conditions and when somatostatin was bath applied. Scale bar corresponds to 40 µm. The data is presented as mean ± standard error.

After the baseline settings were established, we continued by analyzing the effect of somatostatin on the non-spontaneously active Grpr-Cre.GCaMP6 neurons. In intact cord, somatostatin (1 µM) induced activation (mean reaction time of 5.8 ± 0.8 minutes) in 33.8% (25 out of 74 neurons, n = 3 animals) of the non-spontaneously active Grpr-Cre.GCaMP6-expressing neurons (Fig. 5a–c). When somatostatin was applied to coronal slices, 17.1% (24 out of 140 neurons, n = 2 animals) of the non-spontaneously active Grpr-Cre.GCaMP6-expressing neurons (mean reaction time of 5.9 ± 0.9 minutes) showed an increase in baseline fluorescence (Fig. 5d–f, Video S1). The mean *∆F/F* values when somatostatin was applied were 16 ± 2.9% for intact spinal cord and 60.7 ± 9.5% for coronal slices, which represents a significant increase in fluorescence when compared to intact spinal cord (7.9 ± 1.9%) or coronal slices (27.5 ± 2.6%) when the peptide was not bath applied (*p* = 0.0124 and *p* < 0.0001, respectively) (Fig. 5g). The peptide somatostatin was thus able to activate a sub-population of Grpr-Cre.GCaMP6 neurons, results that complements the electrophysiological data and shows that the itch-related Grpr-Cre population is under inhibitory control that is released upon disinhibition induced by somatostatin.

## Discussion

The GRP/GRPR system in the dorsal horn is suggested as a key component of a neuronal network that transmits itch-related information^4,5,26^. Besides transmitting upstream sensory information, GRPR interneurons are proposed to be part of a circuit that is modulated by inhibitory interneurons^7,15^ to prevent spontaneous discharge and consequently the sensation of spontaneous itch. The hypothesis that GRPR dorsal horn interneurons are under local inhibition is so far supported only by pharmacological behavioral experiments. Thus, in the present work we complement the behavioral findings with *in vitro* evidence for local tonic inhibition that controls the activity of the GRPR neurons. We also show that this itch-related population can be activated by disinhibition through somatostatin, thereby demonstrating on a cellular level alternative ways for the induction of itch via the GRPR system, i.e. increased excitation via GRP or decreased inhibition through direct disinhibition of the GRPR population.

### GRPR dorsal horn interneurons are under tonic inhibition

Dorsal horn GRPR-expressing neurons receive evoked glutamatergic input from primary afferent C-fibers^27^. Additionally, our data showed that GRPR neurons received both spontaneous excitatory and inhibitory inputs from local spinal neurons. The spontaneous EPSCs were mostly mediated by AMPA receptors, as the application of CNQX or NBQX blocked 90% of the EPSCs. This was further supported on a transcriptional level, as AMPA receptor subunit *Gria2* was expressed in all *Grpr* neurons and the remaining receptor subunit genes were present in *Grpr* neurons (Table S1). In a recent analysis, where Grp-Cre neurons were driven by optogenetics, light-evoked EPSCs in patched Grpr-*egfp* neurons could be blocked by NBQX^28^, further strengthening the conclusion. CNQX also induced a depolarizing current, which could be due to two different mechanisms. CNQX could have induced depolarization directly through the glutamatergic synapses on GRPR neurons as CNQX can act as a partial agonist for AMPA receptors via one of its auxiliary subunits, TARPs (transmembrane AMPA receptor regulatory proteins)^21^. Also, the excitatory effect of CNQX is independent of its antagonistic action on ionotropic glutamate receptors^29^, which is similar to our observation that both blockages of fast transient currents and depolarization occurred upon application of CNQX. Another possible mechanism is indirectly through inhibitory synaptic inputs by reducing the excitability of the presynaptic inhibitory neurons. However, this possible mechanism was excluded, since another AMPA receptor antagonist NBQX did not induce any depolarization.

**Figure 6:**
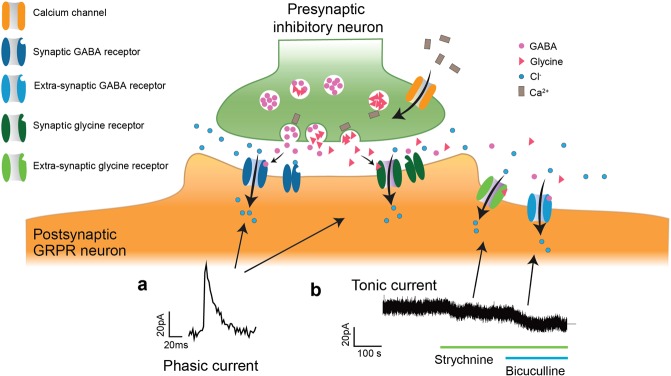
Schematic illustration of phasic and tonic inhibitory currents on spinal GRPR neurons. The action potential-activated presynaptic voltage-gated calcium channel induces calcium influx to the presynaptic terminal, which triggers the release of GABA and glycine. GABA and glycine could be released from different synapses or from a GABA and glycine co-release synapse where the transmitters are released from the same or separated vesicles. Released transmitters bind to the postsynaptic fast-desensitized GABA and glycine receptors on the GRPR neurons and the activation of the receptors induces fast synaptic inhibitory currents. Some transmitters diffuse out of the synaptic cleft and bind to the high affinity peri-/extra-synaptic GABA/glycine receptors^52,53^. The activation of the extra-synaptic GABA/glycine receptors on the postsynaptic GRPR neuron, in contrast to the fast synaptic current (**a**), generates persistent and long-lasting inhibitory currents, which are generally referred to as tonic inhibitory currents (**b**) and are revealed as a depolarization upon the application of the glycine and GABA receptor antagonists strychnine and bicuculline, respectively.

Besides receiving spontaneous excitatory inputs, GRPR neurons are expected to receive spontaneous inhibition as selective ablation/inhibition of dorsal horn inhibitory interneurons results in significantly enhanced scratching responses to pruritic agents or induced spontaneous targeted behavior (licking and biting)^6,9^. Additionally, both inhibitory transmitters GABA^30,31^ and glycine^31^ are involved in itch inhibition. In support of this, we show here that GRPR neurons received both spontaneous glycine and GABA-induced IPSCs and tonic inhibitory currents. In addition to the glycinergic and GABAergic sIPSCs, our data also showed mixed events that accounted for glycine and GABA co-release, which indicated the existence of synapses on GRPR neurons where glycine and GABA were co-released (Fig. 6). The glycine and GABA co-releasing synapses have also been discovered in many regions of the central nervous system^22,32–34^. Expression of GABA (high prevalence of *Gabra3*) receptor subunits’ genes in *Grpr* neurons supports these findings. However, functional glycine receptors are heteropentamers of alpha and beta subunits^35^, and *Grpr* neurons only show strong expression of the beta subunit (*Glrb*), and weak expression of the alpha subunits (*Glra1–4*). The correlation between mRNA levels and protein levels in single cells is weak, and inference of protein levels from scRNAseq data can be misleading. At a single cell level, cells can express mRNA without translating detectable levels of protein; similarly, proteins can be detected in the absence of corresponding mRNAs^36^. Here the latter is likely the case, as the GRPR neurons receive strong glycinergic input concluded from the electrophysiological experiments.

Tonic inhibitory current, in contrast to transient synaptic current, inserts a persistent inhibition (hyperpolarization) through consistently open high affinity peri/extra-synaptic receptors^37,38^ (Fig. 6). Therefore, the chloride ions can freely flow into the cell through the open peri/extra-synaptic receptor channels and hyperpolarize the membrane towards the equilibrium potential of chloride. Depolarization of the membrane, on the other hand, can increase the influx of the chloride ion, which acts as a buffer to neutralize the depolarizing effect of excitatory synaptic inputs and reduce the excitability of the neuron (Fig. 3). Our analysis showed that antagonizing the tonic inhibitory current significantly reduced the rheobase of GRPR neurons, i.e. the GRPR neurons became more excitable. It is worth noticing that the mean difference in the rheobase (32.4 pA) resembles the sum of the mean tonic currents induced by glycine and GABA (32.75 pA), which suggests that the blocking of the tonic currents was the main contributor of the reduced rheobase. Thus, tonic inhibition can interpose a gating effect to the itch signal transmission, which in behavioral experiments, with impaired inhibition, may be the explanation for the observed increase in itch behavior^6,7,9^. Recently, Pagani *et al*. demonstrates that GRPR neurons requires GRP released during repetitive burst-like presynaptic activity (achieved through optogenetic activation of spinal Grp-Cre neurons) to open the gate for itch transmission. Thus, it is tempting to speculate that repetitive burst-like release of GRP is needed due to the persistent inhibition exerted by tonic GABA and glycine currents on GRPR neurons. Reduction of GABAergic tonic current, but not phasic current, is correlated to inflammation-induced thermal hyperalgesia in the ventrobasal thalamus^39^ and enhancing of extra-synaptic GABA receptors attenuates acute nociception in mice^40^, indicating a complex role for tonic inhibition in regulating sensory signaling. Since both glycine and GABA exert tonic inhibition on GRPR cells, our data show the existence of a complex inhibitory system that regulates the excitability of the GRPR neurons, thereby gating the transmission of the itch signal through the GRP/GRPR network. In agreement with our findings, phasic and tonic glycinergic/GABAergic inputs to undefined dorsal horn neurons have been reported^10,12,13^.

### Somatostatin activates itch-related dorsal horn interneurons

Following the hypothesis that spinal cord neurons are under tonic inhibition, impairment of the inhibitory tone would increase local neuronal activity. Indeed, blockage of the inhibitory tone in ventral horn motor neurons by the GABA antagonist bicuculline evokes irregular bursts and when applied together with strychnine regular rhythmic bursts can be observed^41^. In the dorsal horn, disinhibition through blockage of GABA_A_ and glycine receptors is able to increase the neuronal firing rate in cats^42,43^ and rats^44^ and also the c-Fos expression level^45^ in rat dorsal horn neurons. The somatostatin receptor agonist octreotide is known to induce scratch behavior when injected intrathecally and somatostatin has a hyperpolarizing effect on targeted neurons^7,15^. In the dorsal horn, the somatostatin receptor sst_2A_ is expressed mainly in inhibitory interneurons^46^, which indicates that somatostatin induces itch through disinhibition that would represent an alternative way to the induction of itch. Our experiments showed that somatostatin induced activation in 33.8% of the Grpr-Cre-recorded neurons from the intact spinal cord (Video S1), showing that Grpr-Cre neurons are partly under local inhibition downstream of somatostatin-induced disinhibition in the dorsal horn. Additionally, 17.1% of the Grpr-Cre neurons in coronal slices showed somatostatin-induced activation suggesting that although part of the local inputs to the population was potentially mechanically impaired by the slice preparation, remaining inhibition was still present that could be disinhibited by somatostatin.

The Grpr-Cre.GCaMP6 population in the spinal cord shows 5.5% spontaneous activity in the intact spine, which increased when the tissue was sectioned (Video S2). Damaged dendritic trees and/or altered balance between excitatory/inhibitory local input may be the explanation for this observed increase in calcium influx. Furthermore, local interneurons are not the only source of inhibition to dorsal horn neurons. The brainstem rostral ventromedial medulla (RVM) sends direct descending fibers to the spinal cord dorsal horn^47^ and GABAergic RVM neurons are known to control the excitability of dorsal horn interneurons^48^. Thus, although local connections remained intact in our intact spinal cord preparation, loss of descending brainstem inhibition was expected as the spinal cord were separated from the brain, which may explain the presence of spontaneous activity in the recorded neurons in the intact spinal cord at control conditions.

In conclusion, we show that GRPR neurons are under tonic and phasic GABAergic and glycinergic inhibitory control to prevent spontaneous discharge. Furthermore, our findings show that pharmacological impairment of the inhibitory tone by applying the peptide somatostatin was effective in inducing activation in a part of this itch-related population. Taken together, this provides direct *in vitro* evidence for the existence of an inhibitory tone controlling the itch-transmitting sensory neuronal network in the spinal cord and for alternative ways for the induction of itch.

## Methods

### Animals

All animal procedures were approved by the local ethical committee in Uppsala (Uppsala djurförsöksetiska nämnd) and followed the Directive 2010/63/EU of the European Parliament and of the Council, The Swedish Animal Welfare Act (Djurskyddslagen: SFS 1988:534), The Swedish Animal Welfare Ordinance (Djurskyddsförordningen: SFS 1988:539) and the provisions regarding the use of animals for scientific purposes: DFS 2004:15 and SJVFS 2012:26. Both female and male mice were used if not otherwise indicated. Grpr-Cre mice^5^ were crossed with either C57bl/6 or *tdTomato* mice and the offspring were genotyped for the presence of the Grpr-Cre allele and/or the *tdTomato* allele. The following primers were used: Grpr-Cre 5′-gtgcaagctgaacaacagga-3′ (forward), and 5′-ccagcatccacattctcctt-3′ (reverse); *tdTomato* 5′-tgttcctgtacggcatgg-3′ (forward), 5′-ggcattaaagcagcgtatcc-3′ (reverse). The Grpr-Cre allele was kept heterozygous.

### Tissue preparation and immunohistochemistry

To fix the tissue for immunohistochemical analysis, the mice were trans-cardially perfused with 1xPBS followed by 4% PFA (Histolab, Sweden) in 1xPBS. The spinal cords were dissected out and post-fixed in PFA for 24 hours. To prevent the formation of large ice crystals during freezing, the tissue was incubated in a stepwise gradient of sucrose solutions, ending with 30% sucrose overnight. The tissue were mounted in cryostat embedding medium (Bio-Optica, Italy) and snap frozen on dry ice. 20 µm sections were cut using a cryocut 1800 cyrostat (Leica, Germany) and the sections were stored at −20 °C until used.

For immunohistochemical analysis, slides were thawed and dried for 15 minutes, and then washed in base buffer three times for 5 minutes. PBS or TBS was used as base buffer depending on the primary antibody (see below). To prevent unspecific binding of the primary antibodies, sections were incubated for 1 hour in blocking solution (0.25% gelatin and 0.5% Triton-X100 in base buffer) at room temperature. Primary antibodies: rabbit anti-PAX2 (Nordic biosite, 901001, 1:200 in TBS-based blocking), guinea pig anti-TLX3 (gift from Prof. Dr. Carmen Birchmeier, 1:1000 in PBS-based blocking) and sheep anti-EBF2 (ThermoFisher, PA5–47890, 1:1000 in PBS-based blocking) were incubated on the slides for 24 hours in 4 °C. Sections were washed three times for 5 minutes in blocking solution, and then incubated with secondary antibody mix, donkey anti rabbit alexa 647 (Invitrogen, A31573, 1:500) or donkey anti sheep alexa 647 (Invitrogen, A21448, 1:500) in blocking solution for 2 hours. Sections were washed three times for 5 minutes in base buffer, and then mounted in ProLong® Diamond Antifade Mountant with DAPI (Life Technologies, USA).

### Imaging and post processing

Wide field images were taken using an Olympus BX61WI fluorescence microscope (Olympus, Japan) on 20 µm cryo-sectioned tissue. Brightness and contrast were adjusted to balance the channels and background subtraction (ImageJ 1.51k, USA) was run on each image.

### Cell counting

Cells were counted in the specified spinal cord lamina^49^ and overlap was analyzed using CellProfiler^50^. Briefly, primary objects were identified for DAPI (nuclei), GFP (Grpr-Cre) and Alexa 647 (IHC) channels. Threshold was automatically set using the minimum cross entropy method in CellProfiler, and the threshold correction factor was manually set to optimize the detection of objects from different IHC runs. Filtering of objects was used to obtain information of Grpr-Cre overlap with IHC. For all settings please review the sample script that can be found as Supplementary data.

### Viral injection

Male and female mice (2–6 months old) were used for experiments based on viral injections. The animals were anesthetized with isoflurane (Baxter, induction at 4%, continuous anesthesia at 2%) and the body temperature was monitored and maintained at 36–37 °C under the procedure using a heating pad (CMA, Sweden). The fur on the back was shaved and a 1 cm incision was made along the midline above vertebrae T12–13 followed by a second incision that went through the connective tissue covering these two vertebrae. Next, small cuts were made into the muscle on each side of L1 vertebra and a clamp was inserted to secure the spinal column. The ligamentum flavum connecting L1 with T13 was cut to reveal the spinal cord. Bilateral injections with either 0 or 20 degrees angle of 500 nl virus each were made into the spinal cord parenchyme 0.4 mm of the midline and 0.5 mm ventral using a 10 µl Nanofil Hamilton syringe (WPI, USA) with a 34 G beveled needle (WPI, USA) guided by a micro syringe pump controller (WPI, USA) mounted on a stereotaxic frame. The tissue was kept moist throughout the procedure by continuous application of sterile saline (9 mg/ml, Fresenius Kabi, Sweden) on the surgical area. AAV2-EF1a-DIO-EYFP (lot number AV4842D, titer: 4.6*10^12^ vg/ml) was purchased from UNC Vector core and AAV9-CAG-DIO-GCaMP6f-WPRE (lot number CS0722, titer 1.52*10^13^ vg/ml) from UPenn Vector core. Bupivacaine (Marcain; AstraZeneca, 2 mg/kg) was administered as a local anesthetic at the site of surgery and buprenorphine (Vetergesic Vet; Orion Pharma, 0.1 mg/kg) was used for postoperative analgesia. The animals were left 2–10 weeks after surgery before use in any experiments to achieve full recovery and efficient expression of virally delivered proteins.

Injection for IHC overlap analysis was performed as described above, but with the following differences: 100 nl of AAVDJ-EF1a-DIO-HTB (lot date 2017, titer 1.83*10^11^ vg/ml) produced by the Salk Institute GT3 (Gene Transfer, Targeting, and Therapeutics) core facility was injected 0.4 mm of the midline and 0.5 mm ventral at a speed of 50 nl/minute. The animals were sacrificed 10 days after injection.

### Electrophysiology and calcium imaging

For patch clamp recording, t*dTomato*;Grpr-Cre mice (age from 6 weeks to 5 months, the age was randomly distributed and not biased to a specific set of experiments) were anesthetized with ~0.5 ml isoflurane (FORANE, Baxter, USA) for 1–2 min and injected with 0.3 ml Ketamin (Ketalar, 10 mg/ml, Pfizer) and 0.3 ml Medetomidine (Domitor, 1 mg/ml, Orion Pharma). After the anaesthetization, the mouse was perfused with NMDG-HEPES based cutting solution composed of (mM): 93 N-methyl-D-glucamine, 2.50 KCl, 1.20 NaH_2_PO_4_, 30 NaHCO_3_, 20 HEPES, 25 Glucose, 5 sodium ascorbate, 2 Thiourea, 3 sodium pyruvate, 10 MgSO_4_.7H_2_O, 0.5 CaCl_2_.2H_2_O. After perfusion, the ventral part of the vertebral column of the mouse was cut and removed to expose the spinal cord. The spinal cord was isolated from the vertebra and dura layers were carefully removed. The isolated spinal cord was then cut into 350 µm thick transverse (coronal) slices with vibratome (Leica vt1200) in the cold (4 °C) continuously oxygenated (95% O_2_ and 5% CO_2_) the NMDG-HEPES based cutting solution (same as above).

Alternatively, virus-injected (calcium imaging) Grpr-Cre adult mice (2 to 6 months old, the age was randomly distributed and not biased to a specific set of experiments) were perfused and their spinal cord dissected and sliced in 300 µm in the sagittal, coronal or horizontal plane or kept intact in cold NMDG-HEPES based recovery solution. The cut slices were then recovered in aCSF (concentration in mM: 126 NaCl, 2.5 KCl, 1.25 NaH_2_PO_4_, 26 NaHCO_3_, 10 glucose, 1.5 CaCl_2_, 1.5 MgCl2) at 36 °C for 1 hour and in room temperature for a minimum of 30 minutes before they were placed in the recording chamber. The slices were then transferred to a recording chamber, where Grpr-Cre neurons were identified using a 60x or 20x water-immersion objective (LUMPlan FI, 0.90 numerical aperture (NA), Olympus) through either red fluorescent protein for patch clamp or green fluorescent protein for calcium imaging, visualized on a Zyla sCMOS camera (Andor Technology Ltd) connected to a green (550 nm) or blue (490 nm, CoolLED system) fluorescent LED light source. Patch electrodes (6–12 MΩ) from borosilicate glass capillaries (GC150F-10 Harvard Apparatus) pulled on a PC-10 gravitational pipette puller (Narishige) contained a K^+^ based internal solution (in mM): 120 K-gluconate, 40 HEPES, 1.02 MgCl_2_, 2.17 MgATP, 0.34 NaGTP, with pH adjusted to 7.2 using 1 M KOH with an osmolarity of 266 mOsm/L. Liquid junction potential was calculated corrected before each patched neuron.

Whole cell patch clamp recordings were made using a multiclamp 700B amplifier (Axon Instruments) and digitalized with Digidata 1440 A (Molecular Devices), low pass filtered at 10 kHz, digitized at 20 kHz, and acquired/analyzed in WinWCP software (Dr. J. Dempster, University of Strathclyde, Glasgow, UK), Clampfit 10.3 (Molecular devices, USA), Mini Analysis (Synaptosoft, USA) and Matlab (Mathworks). Grpr-Cre neurons were first identified with the help of red fluorescence (*tdTomato*). When the whole cell configuration was achieved, action potentials were induced by current step from 0 to 150 pA with an increment of 10 pA (pulse duration 500 ms) to monitor the viability of the patched neuron and the rheobase was determined by using 1 pA increment current step (pulse duration 500 ms). Then the neuron was held in voltage clamp mode. Different clamping voltages were chosen for studying spontaneous excitatory postsynaptic currents (sEPSCs) and spontaneous IPSCs (sIPSCs). For measuring the sEPSCs, the cell membrane was held at −70 mV (the reversal potential of IPSC), while holding voltage of 0 mV (the reversal potential of EPSC) was used for sIPSCs measurement. Minimum 5 minutes of stable baseline was used for control, which was followed by the application of the desired compounds. In the sEPSCs measurement, 25 µM of AMPA/kainate receptor antagonist 6-cyano-7-nitroquinoxaline-2,3-dione (CNQX, Sigma) was applied via perfusion system after stable baseline was established. Alternatively, 10 µM of AMPA/kainite receptor antagonist 2,3-Dihydroxy-6-nitro-7-sulfamoyl-benzo(F)quinoxaline (NBQX, Sigma) was also tested as a comparison to CNQX. For studying the sIPSCs, 4 µM strychnine (Sigma) and 20 µM bicuculline (Sigma) were used to block glycine receptors and GABA receptors respectively. To determine the effect of the inhibitory inputs on the excitability of the GRPR neurons, slices were continuously perfused with 4 µM strychnine and 20 µM bicuculline. At the end of every recording, 300 nM GRP (Gastrin-Releasing Peptide, Phoenix Europe GmbH) was applied to confirm the expression of the GRP receptors on the patched cells, which was indicated by the depolarization current upon the application of GRP. Only the GRPR positive neurons were included in the statistics. Every sEPSC/sIPSC event was detected and analyzed by Mini Analysis software. The time points of the events were used to calculate sEPSCs/sIPSCs frequency and decay time constant values were used for analyzing the event kinetics to distinguish glycinergic and GABAergic sIPSCs^22^.

Spontaneous calcium imaging activities from superficial dorsal horn Grpr-Cre AAV9-CAG-DIO-GCaMP6-infected neurons, and from superficial Grpr-Cre AAV9-CAG-DIO-GCaMP6-infected neurons in the presence of bath applied somatostatin (1 µM, Tocris), were recorded through a blue LED excitation light and signals were acquired in a frame rate of 2 Hz. Baseline spontaneous activities were monitored in 5 minutes recordings followed by a new protocol where applied somatostatin reached the recording chamber after 1 minute of each recording. The total recording time in each experiment was 15 minutes. Data presented here shows relative change in the fluorescence signal from a region of interest (ROI). *∆F/F* was calculated based on the mean value of baseline activity.

### Statistics

Cells were counted for each dorsal horn and reported as a mean ± standard error of the mean. Prevalence of spontaneous Grpr-Cre activation was calculated as a proportion of neurons with *∆F/F* value above 3.5% in relation to all Grpr-Cre.GCaMP6.*gfp*-expressing neurons in each set of experiments. The value of 3.5% was based on simultaneous current clamp and calcium-imaging experiment, where action potential-induced calcium signals could clearly be detected and distinguished from the background, while any detected calcium signals lower than 3.5% were considered as noise and not included in the statistics. Since only the bursting action potential-triggered calcium influx was strong enough to induce a clearly detectable calcium signal, every detected GCaMP6.*gfp* signal represented a bursting activity. Thus, neuronal activity was considered as repeated bursting if consisting of at least three peaks of calcium activities distributed in 5 minutes of recording.

*∆F/F* values are presented as mean ± standard error of the mean and mean values were statistically compared using Mann-Whitney U-test (GraphPad Software, Inc., San Diego, CA).

The electrophysiological data values are presented as mean ± standard error of the mean and the mean values were statistically compared using two-tailed student’s T-test (GraphPad Software, Inc., San Diego, CA).

### Single-cell data processing and analysis of glutamatergic and GABAergic *Grpr*-expressing neurons

The Häring *et al*. (2018) Supplementary Table 1 dataset containing 24,384 genes from 1,545 spinal dorsal horn neurons^8^ was accessed through Gene Expression Omnibus (GEO: GSE103840). The dataset was pre-processed and analyzed with SCANPY 1.3.1^51^ using Python 3.7.0. The dataset was normalized by setting the number of counts per cell to the median counts per cell (SCANPY, normalize_per_cell) and the counts were thereafter logarithmized (SCANPY, log1p). All neurons expressing *Grpr* (normalized expression > 0.01) were isolated from the dataset and the gene expression levels of excitatory/inhibitory marker genes and receptor subunits important for GABAergic, glutamatergic and glycinergic signaling input were visualized (SCANPY, stacked_violin). The expression prevalence (gene expressed if normalized expression < 0.01) was thereafter calculated for the selected genes in the *Grpr* population.

In the supplementary information, the normalized mean expression of the selected genes in the Häring *et al*. molecular subtypes (Glut1–15, Gaba1–15) (SCANPY, matrixplot) and in the *Grpr*-expressing (normalized expression > 0.01) molecular subtypes (SCANPY, stacked_violin) are visualized. Full code is available at https://github.com/JonETJakobsson/Grpr-input.

## Supplementary information

Supplementary material

Video S2

Video S1
