# Supplementary material for: Spinal gastrin releasing peptide receptor expressing interneurons are controlled by local phasic and tonic inhibition
Source: Sci Rep. 2019 Nov 12;9:16573. doi: 10.1038/s41598-019-52642-3 (PMC6851355; doi:10.1038/s41598-019-52642-3)
Supplement: Supplementary file 1 — Supplementary material [file 41598_2019_52642_MOESM1_ESM.docx]

**Spinal gastrin releasing peptide receptor expressing interneurons are controlled by local phasic and tonic inhibition**

Fabio B. Freitag, Aikeremu Ahemaiti, Jon E.T. Jakobsson, Hannah M. Weman and Malin C. Lagerström

Department of Neuroscience, Uppsala University, Uppsala, Sweden

**Supplementary material**


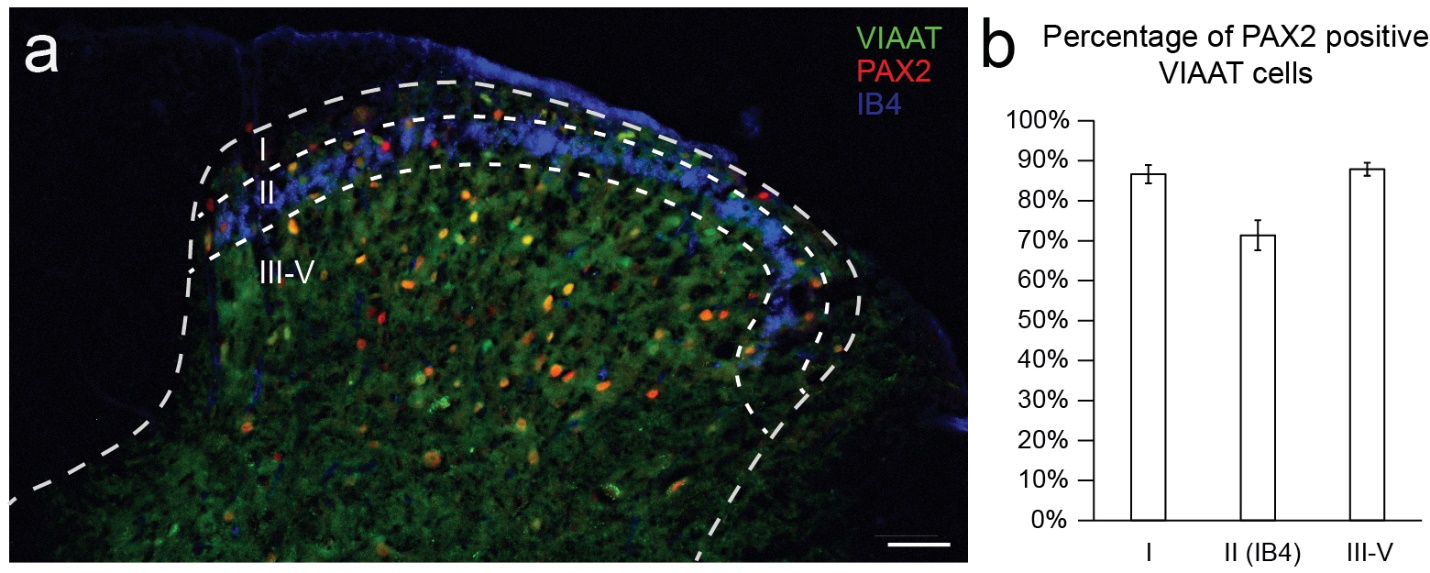


**Figure S1. *Viaat*-EGFP cells co-express the inhibitory marker PAX2.** **(a)** Coronal section of the right dorsal horn of a *Viaat*-EGFP animal. *Viaat*-EGFP cells in green, PAX2 cells in red and IB4 layer in blue. Scale bar is 100µm. **(b)** Percentage of *Viaat*-EGFP positive cells that co-express PAX2 grouped by lamina I, II and III-V. Error bars are SEM.

**
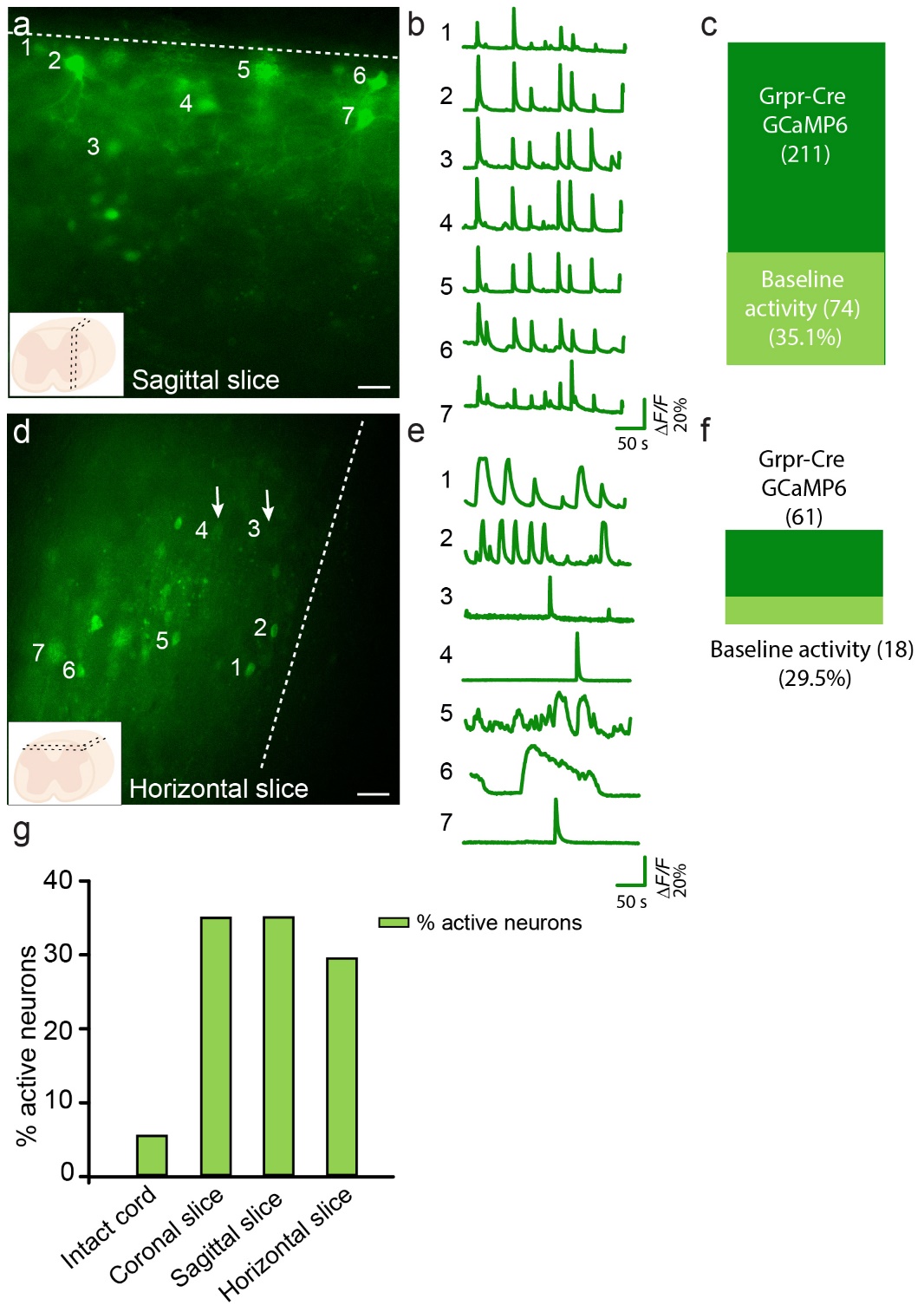
**

**Figure S2.** The angel of the slice preparation does not influence the proportion of spinal Grpr-Cre neurons expressing GCaMP6 that show basal activity. Example traces from representative samples are shown. **(a)** 300 µM sagittal slice with Grpr-Cre neurons expressing GCaMP6. Numbers 1 – 7 indicate seven different neurons with spontaneous activity. **(b)** Fluorescence change traces from the seven neurons indicated by 1 – 7 in **a**. **(c)** Proportion of Grpr-Cre neurons expressing GCaMP6 in sagittal slices that showed spontaneous activity. In total 35.1% (74 out of 211 neurons, n = 3 animals) of the Grpr-Cre.GCaMP6 neurons showed increased basal activity in section prepared in the sagittal plane. **(d)** 300 µM horizontal slice with Grpr-Cre neurons expressing GCaMP6. Numbers 1 – 7 indicate seven different neurons with spontaneous activity**. (e)** Fluorescence change traces from the seven neurons indicated by 1 – 7 in **d.** **(f)** Proportion of Grpr-Cre neurons expressing GCaMP6 in horizontal slices that showed spontaneous activity. In total 29.5% (18 out of 61 neurons, n = 2 animals) of the Grpr-Cre.GCaMP6 neurons showed increased basal activity in section prepared in the horizontal plane. **(g)** Summary of prevalence in the level of basal neuronal activation when the spinal cord was imaged in different experimental preparations (intact cord, coronal, sagittal and horizontal slices). For visualization of spontaneous activity displayed by the Grpr-Cre.GCaMP6 population, please see Video S2. Scale bar corresponds to 40 µm.

**
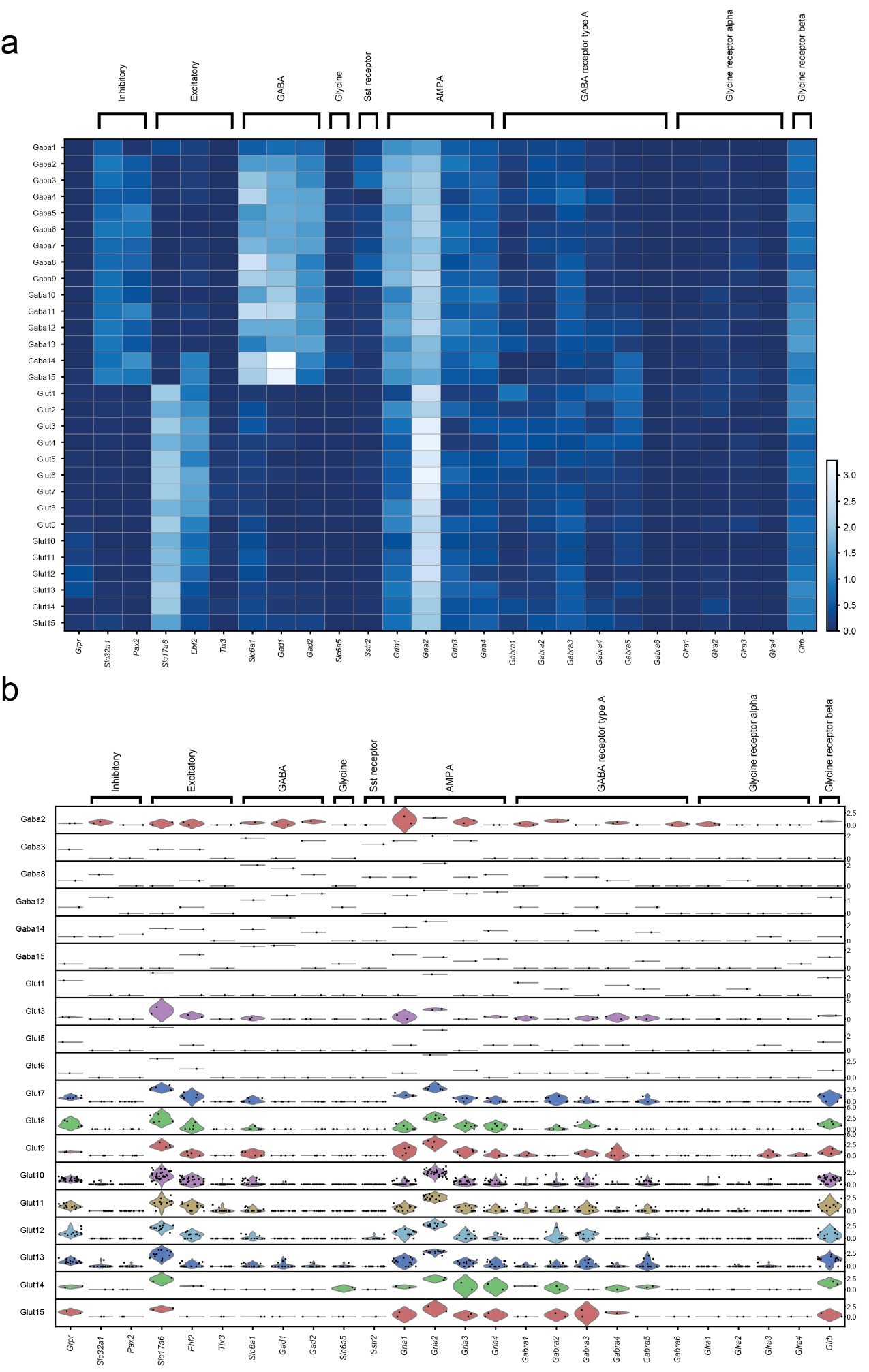
**

**Figure S3. *Grpr* is mainly expressed in the Glut10-13 Häring *et al* molecular subtypes.** **(a)** Matrix plot of logarithmized target gene expression (log1p) in the Häring *et al* molecular subtypes. By examining the expression of marker genes in the Häring et al. molecular subtypes, it was seen that *Grpr* was mainly expressed in the excitatory Glut10-13 cell types, but could also be detected in other excitatory subtypes. The excitatory subtypes Glut7-12 were all superficial populations expressing *Ebf2* while Glut13 defined a cluster of deeper interneurons, which also overlapped with *Ebf2* (Häring et al. 2018). In our histological findings, we did not see any EBF2 antigen expression in the deeper laminae, indicating that although the *Ebf2* mRNA was expressed in Glut13 neurons (Häring et al. 2018), the protein might not have been translated. Correlating gene expression and corresponding protein level is a difficult task since many biological factors can influence this correlation (Maier et al. 2009). Expression of *Tlx3* was found in the excitatory Glut7-8, Glut10-11 and Glut14 cell types and did overlap with *Ebf2* expression to a considerable extent. Also, *Tlx3* was expressed in Glut14, which did not have *Ebf2* expression, hence TLX3 and EBF2 labeled partially overlapping excitatory cell populations. **(b)** Stacked violin plot of logarithmized target gene expression (log1p) in *Grpr*-expressing (log1p > 0.01) Häring *et al* molecular subtypes.

**Table S1.** **The prevalence of marker genes, and GABAergic, glutamatergic and glycinergic receptor subunits in the *Grpr*-expressing population from Häring *et al* dataset.** Genes considered prevalent if logarithmized expression > 0.01.

| Function | Genes | Percentage of *Grpr* neurons expressing gene [%] |
| --- | --- | --- |
|  |  |  |
| Inhibitory marker | *Pax2* | 1.9 |
|  | *Slc32a1* | 8.7 |
|  |  |  |
| Excitatory marker | *Ebf2* | 64.1 |
|  | *Tlx3* | 2.9 |
|  | *Slc17a6* | 94.2 |
|  |  |  |
| GABA_A_ receptor subunits | *Gabra1* | 14.6 |
|  | *Gabra2* | 23.3 |
|  | *Gabra3* | 44.7 |
|  | *Gabra4* | 13.6 |
|  | *Gabra5* | 27.2 |
|  | *Gabra6* | 1 |
|  |  |  |
| AMPA receptor subunits | *Gria1* | 62.1 |
|  | *Gria2* | 100 |
|  | *Gria3* | 61.2 |
|  | *Gria4* | 38.8 |
|  |  |  |
| Glycine receptor subunits | *Glra1* | 2.9 |
|  | *Glra2* | 4.9 |
|  | *Glra3* | 4.9 |
|  | *Glra4* | 1.9 |
|  | *Glrb* | 78.6 |
| Somatostatin receptor 2 | *Sstr2* | 2.9 |

**Supplementary sample script**

Sample script can be used with CellProfiler to replicate the counting approach used in this study. Images should contain three channels; one for nuclei, one for cell of interest and one for the IHC. Proper object detection should be ensured by manually setting the threshold correction factor for each channel for each experiment. Object size will depend on the resolution of images, and on the size of the cells under investigation. This parameter will have to be estimated by the user.

**Video S1.** Grpr-Cre neurons expressing GCaMP6 show induced activation by somatostatin in both intact spinal cord and coronal slices. The video is on repeat.

**Video S2.** Grpr-Cre neurons expressing GCaMP6 show an increase in spontaneous activity in coronal, sagittal and horizontal slices when compared to intact spinal cord. The video is on repeat.
